# Unveiling Antibiotic Resistance in Ventilator-Associated Pneumonia Patients: A Comprehensive Analysis

**DOI:** 10.7759/cureus.104484

**Published:** 2026-03-01

**Authors:** Arti Agrawal, Pragya Shakya, Parul Garg, Ajeet S Chahar, Dharmendra Kumar, Ayushi Gupta, Yogita Singh

**Affiliations:** 1 Microbiology, Sarojini Naidu Medical College, Agra, IND; 2 Medicine, Sarojini Naidu Medical College, Agra, IND; 3 Ear, Nose and Throat, Sarojini Naidu Medical College, Agra, IND; 4 Liberal Arts and Humanities, Jindal Global University, Sonipat, IND; 5 Biotechnology, Raja Balwant Singh College, Agra, IND

**Keywords:** endotracheal aspirate, icu, multidrug resistant organisms, vap, ventilator-associated pneumonia

## Abstract

Background: Ventilator-associated pneumonia (VAP) remains a major healthcare-associated infection, especially in resource-limited settings where multidrug-resistant organisms are prevalent. This study assessed the bacteriological profile and antimicrobial susceptibility patterns of endotracheal aspirates (ETA) from clinically suspected VAP patients in a tertiary care ICU, and compared microbial distribution between early and late-onset VAP.

Methods: A descriptive cross-sectional study was conducted from October 2024 to September 2025 in a tertiary care teaching hospital. Adult patients who were mechanically ventilated for more than 48 hours and met the modified Clinical Pulmonary Infection Score (CPIS) criteria (score >6) were included. ETAs were analyzed using semi-quantitative culture methods, with ≥10⁵ CFU/mL considered significant, and antimicrobial susceptibility testing was performed in accordance with CLSI 2023 guidelines. Demographic variables, risk factors, bacterial isolates, and resistance patterns were analysed using chi-square tests.

Results: Among 596 ventilated patients, 195 (incidence proportion 32.7%) had significant culture-positive VAP. The incidence density was 33.6 per 1000 ventilator-days. Early-onset VAP accounted for 57.9% (113/195) and late-onset VAP for 42.1% (82/195). No significant association was observed between VAP onset and age, sex, or indication for intubation. Gram-negative pathogens predominated in both groups (76.1% early vs. 78% late). *Acinetobacter baumannii* was the most common isolate (35.3% early, 36.2% late), followed by *Klebsiella pneumoniae* and *Pseudomonas aeruginosa*. A significant difference was noted in Gram-negative distribution between early and late VAP (p=0.009). Extensive multidrug resistance was observed, with universal susceptibility only to polymyxin B and colistin. Carbapenem susceptibility varied by pathogen, with *A. baumannii* showing higher sensitivity in late-onset cases.

Conclusion: VAP burden in this ICU remains high, with Gram-negative multidrug-resistant organisms, particularly *A. baumannii* and *K. pneumoniae, *driving most infections. The findings underscore the urgent need for strengthened antimicrobial stewardship, routine resistance surveillance, and strict adherence to VAP prevention bundles. Early culture-guided de-escalation is crucial to optimize outcomes and curb resistance.

## Introduction

Healthcare-associated infections (HAIs) are a cause of significant concern. Among HAIs, ventilator-associated pneumonia (VAP) is one of the most common infections, which occurs in patients who have been on mechanical ventilation for more than 48 hours. In critical care ICUs, it contributes to higher morbidity, longer ICU stays, higher healthcare costs, and higher mortality rates [1].

Pathogenesis of VAP involves colonization of the lower respiratory tract with pathogenic microorganisms. Factors like compromised host defences, endotracheal intubation, and secretory micro aspiration promote this pathogenesis [2].In resource-constrained areas, endotracheal aspirate (ETA) is still a popular cost effective and less intrusive diagnostic technique which allows reliable identification of pathogenic organisms and assessment of their antibiotic susceptibility, directing appropriate empirical and targeted therapy [3].

The International Nosocomial Infection Control Consortium has found the incidence of VAP in developed countries as high as 13.6/1000 mechanical ventilation (MV) days. The average VAP rates reported in India range from 8.9 to 46 VAP episodes per 1000 MV. VAP rates have been found to be significantly higher in Asian countries, as compared to Western countries. This diversity emphasizes the necessity of region-specific surveillance in informing infection prevention efforts [4]. In India, ICU pneumonia has a crude death rate of 67.4%, with approximately 40% attributed to infection alone [5].

The clinical pulmonary infection score (CPIS) is commonly used for the diagnosis of VAP. The score is calculated using six parameters: temperature (36.5-38.4°C = 0, 38.5-38.9°C = 1, ≥39°C or ≤36°C = 2); leukocyte count (4,000-11,000/mm³ = 0, <4,000 or >11,000/mm³ = 1, <4,000 or >11,000/mm³ with band forms ≥50% = 2); tracheal secretions (rare = 0, abundant = 1, abundant and purulent = 2); oxygenation assessed by PaO₂/FiO₂ ratio (>240 or presence of ARDS = 0, ≤240 without ARDS = 2); chest radiograph findings (no infiltrate = 0, diffuse or patchy infiltrate = 1, localized infiltrate = 2); and tracheal aspirate culture results (negative or light growth = 0, moderate or heavy growth = 1, moderate/heavy growth with same organism on Gram stain = 2). Each variable is scored from 0 to 2, with a maximum total score of 12. A CPIS greater than 6 suggests a high likelihood of VAP, whereas a score of 6 or less makes pneumonia less likely [6].

The rise of multidrug-resistant (MDR), extensively drug-resistant (XDR), and even pan-drug-resistant (PDR) pathogens in ICU settings has made management even more difficult [7]. *Pseudomonas aeruginosa, Acinetobacter baumannii, Klebsiella pneumoniae,* and *Staphylococcus aureus* are commonly implicated in VAP. They exhibit increasing resistance in areas with high antibiotic pressure and inadequate infection management policies [8,9]. Studies of bacterial flora and their resistance profile differ with location and timing. Therefore, local monitoring helps in tailoring the antimicrobial resistance reduction stewardship efforts and improving patient outcomes.

In the current study, we have assessed the bacteriological profile and antibiotic susceptibility patterns of endotracheal aspirates from clinically suspected VAP patients from a tertiary care hospital's ICU. We also attempted to investigate any variations in microbial distribution between early and late-onset VAP.

## Materials and methods

A cross-sectional study was conducted over a period of one year (October 2024- September 2025) in the Department of Microbiology and the Medical Intensive Care Unit (MICU) of Sarojini Naidu Medical College, Agra, a tertiary care teaching hospital. Patients with clinical suspicion of ventilator-associated pneumonia (VAP) were prospectively enrolled. Ethical approval was obtained from the Institutional Ethics Committee prior to study initiation (SNMC/IEC/DHR/2024/03 dated 6th September 2024, and written informed consent was obtained from all participants or their legally authorized representatives.

Study population and diagnostic criteria

All patients aged ≥18 years who were mechanically ventilated for >48 hours and clinically suspected of developing VAP based on CPIS criteria were eligible for inclusion. Patients with pneumonia prior to mechanical ventilation or within 48 hours of mechanical ventilation developing VAP were excluded from the study. CPIS at baseline was assessed based on the first five variables, i.e., temperature, blood leukocyte count, tracheal secretions, oxygenation, and character of pulmonary infiltrate. The CPIS at 72 hours was calculated using all seven variables, incorporating radiographic progression of pulmonary infiltrates and the culture results of tracheal aspirates. A score greater than 6 at baseline or at 72 hours was considered indicative of ventilator-associated pneumonia. Further patients were grouped into:

Early-onset VAP: when VAP occurs <4 days after intubation

Late-onset VAP: when VAP occurs ≥4 days after intubation

Clinical parameters, potential risk factors, and outcomes were documented from medical records and bedside charts. From each patient, the demographic data and primary diagnosis, co‐morbidities, date of admission in hospital and ICU were noted. The study patients were monitored every third day for the development of VAP using clinical and microbiological criteria until either discharge or death. The relevant data were recorded from medical records, bedside flow sheets, radiographic reports, and reports of microbiological studies of the patients.

Collection of endotracheal aspirates (ETA) 

ETA (≥1 mL) was collected under strict aseptic precautions after 48 hours of intubation whenever VAP was suspected. Sampling was performed using a 22-inch Ramson’s 12F suction catheter with a mucus extractor, inserted 25-26 cm into the endotracheal tube. In cases of low secretion volume, chest vibration or percussion was applied for 10 minutes to facilitate retrieval. Tracheal aspirates were classified as absent, non-purulent, or purulent by an experienced senior nursing attendant who cared for the patients daily. The ETA sample was immediately transported to the microbiology laboratory.

Microbiological processing

ETA was subjected to Gram staining and semiquantitative culture based on clinical suspicion. Quantitative cultures are used to diagnose VAP in endotracheal aspirates with a threshold of ≥10^5^ CFU/ml. Each dilution was inoculated onto sheep blood agar, chocolate agar, and MacConkey agar using a calibrated Nichrome loop (0.01 mL; Hi-Media, Mumbai, India). Plates were incubated at 37°C for 18-24 hours, then CA was incubated in 5% CO₂. Bacterial growth of ≥10⁵ CFU/mL was deemed significant, while growth below this level was considered colonization or contamination. The culture media employed were Blood, Chocolate, and MacConkey agar. Bacterial identification was carried out by applying standard microbiological techniques. Antimicrobial susceptibility testing was done using the Kirby-Bauer disc diffusion method, which was interpreted in accordance with the Clinical and Laboratory Standards Institute (CLSI) 2023 recommendations [10]. After 48 hours, both the culture and antibiotic susceptibility test results were reported. *Escherichia coli* ATCC 25922 and *Pseudomonas aeruginosa *ATCC 27853 were used as quality control strains. Isolates showing reduced susceptibility to imipenem were screened for metallo-β-lactamase (MBL) production using the imipenem-EDTA combined disc test. Broth microdilution was performed for colistin and polymyxin B susceptibility testing. 

Statistical analysis

Data were analysed using descriptive statistics. Categorical variables were compared using the chi-square test, with a p-value <0.05 considered statistically significant. The incidence of VAP was calculated using both the incidence proportion per ventilator days and the incidence density of VAP per 1000 ventilator days. The aetiology was presented as percentages.

## Results

A total of 596 patients were on mechanical ventilators during the study period; among them, 401 were clinically suspected of VAP with CPIS >6 after 48hours of MV. Endotracheal aspirate of these 195 patients’ sample had significant bacterial culture growth, which was further analysed. Incidence proportion of VAP was 32.7 per ventilator days. The total duration of MV was 13997.6 days. The incidence density of VAP was 33.6 per 1000 ventilator days. Out of the 195 VAP cases, 113(57.9%) were categorized under early onset VAP and 82 (42.1%) late onset VAP based on predefined duration. The baseline characteristics and risk factors are listed in Table 1.

**Table 1:** Demographics and clinical risk factors of patients grouped as per onset of VAP.

	Variables	Early VAP (N=113)	Late VAP (N=82)	Chi-square test	p-value
Sex	Male	70 (62%)	41 (50%)	2.7659	p-value 0.0963, not significant at >0.05
	Female	43 (38%)	41 (50%)		
Age (in years)	10 to 30	35 (31%)	21 (25.65%)	0.8424	p-value 0.3587, not significant at >0.05
	31 to 60	42 (37%)	35 (42.6%)		
	>60	36 (31.8%	26 (31.7%)		
Cause of intubation	Respiratory Disease(+)	35 (30.9%)	32 (39.2%)	1.3657	p-value 0.2426, not significant at p>0.05
	Respiratory Disease(-)	78 (69.02%)	50 (60.9%)		
	Gastrointestinal Disease (+)	30 (26.5)	15 (18.2%)	1.8246	p-value 0.1768, not significant at p>0.05
	Gastrointestinal Disease(-)	83 (73.3%	67 (81.7%)		
	Neurological Disease(+)	28 (24.7%)	19 (23.17%)	0.0671	p-value 0.7956, not significant at p>0.05
	Neurological Disease(-)	85 (75.2%)	63 (76.82)		
	Injury(+)	13 (11.5%)	8 (9.7%)	0.1511	p-value 0.6975, not significant at p>0.05
	Injury(-)	100 (88.4%)	74 (90.2%)		
	Gynaecological Problems (+)	7 (6.1%)	7 (8.5%)	0.3911	p-value 0.5317, not significant at p>0.05
	Gynaecological Problems (-)	106 (93.8%)	75 (91.4%)		
	Cardiovascular Disease (+)	0 (0%)	1 (1.2%)	Data is insufficient to calculate p-value	
	Cardiovascular Disease (-)	113 (100%)	81 (98.2%)		

Among the 195 VAP patients, 113 (57.9%) had early-onset VAP and 82 (42.1%) had late-onset VAP. Sex and age distribution showed no significant association with the onset of pneumonia. The causes of intubation also did not differ significantly between early- and late-onset VAP.

Overall, none of the demographic characteristics or clinical intubation-related factors demonstrated a statistically significant association with the onset of VAP. A total of 113 bacteria were isolated in early VAP and 82 in late VAP. Among early-onset VAP cases, 86 isolates (76.1%) were Gram-negative, while 64 isolates (78%) were Gram-negative in late-onset VAP. Gram-positive organisms constituted 27 isolates (23.8%) in early-onset and 18 isolates (22%) in late-onset VAP. Statistical analysis using the Chi-square test showed no significant difference in the distribution of Gram-negative versus Gram-positive organisms between early- and late-onset VAP (χ² =0.101, p-value=0.7506).

The distribution of Gram-positive bacterial isolates among early-onset and late-onset VAP cases is summarized in Table 2.

**Table 2:** Distribution of bacterial isolates between early and late-onset VAP. (MR-CoNS): methicillin-resistant coagulase-negative staphylococci.

Micro-organism		Early-onset VAP	Late onset VAP	Chi-square test	p-value
Gram negative	Acinetobacter baumannii	40 (35.3%)	30 (36.2%)	6.8156	p-value 0.009. Significant at p < 0.05
	Citrobacter freundii	2 (1.76%)	3 (3.6%)		
	Klebsiella pneumoniae	29 (25.6%)	14 (17%)		
	Pseudomonas aeruginosa	15 (13.2%)	14 (17%)		
	Enterobacter	0 (0%)	3 (3.6)		
Gram positive	MR-CoNS	1 (0.88%)	2 (2.4%)	1.9444	p-value 0.163, not significant at p >0.05
	*Enterococcus* spp.	6 (5.3%)	6 (7.3%)		
	Staphylococcus aureus	20 (17.6%)	10 (12.1%)		
Total isolates		113	82	195	

*S. aureus* was more frequent in early-onset (17.6%) than late-onset VAP (12.1%). MR-CoNS isolation did not differ significantly between groups ( p-value=0.163). *Enterococcus spp.* were found equally in both groups (6 isolates each). Overall, no statistically significant variation was observed in the Gram-positive pathogen profile between the two categories of VAP.

Among gram-negative isolates, *Acinetobacter baumannii *was the most common pathogen in both groups, identified in 40 (35.3%) early-onset and 30 (36.2%) late-onset VAP cases.* Klebsiella pneumoniae* was detected in 29 (25.6%) early-onset cases versus 14 (17%) late-onset cases, while *Pseudomonas aeruginosa* accounted for 15 (13.2%) and 14 (17%) isolates, respectively.* Citrobacter freundii *was isolated in 2 (1.76%) early-onset and 3 (3.6%) late-onset cases, and* Enterobacter spp.* appeared only in the late-onset group (3 isolates; 3.6%). A statistically significant association was found between the distribution of gram-negative isolates and VAP onset (χ²=6.8156, p-value=0.009), demonstrating that the pathogen profile differed significantly between early- and late-onset VAP at the p-value <0.05 level.

Among the 20 *Staphylococcus aureus* isolates in early VAP and 14 in late VAP, resistance to commonly used β-lactams was frequent, with only 40% susceptibility to cefoxitin, penicillin, piperacillin, meropenem, and cefepime. Conversely, higher susceptibility was observed to tetracyclines (75% early, 71.4% late) and fluoroquinolones (70% early, 57% late). Aminoglycosides (amikacin/high-level gentamicin) retained excellent activity, with 80% and 50% susceptibility in early and late VAP, respectively.

*Enterococcus spp*. (N=6 each for early and late VAP) exhibited limited susceptibility to penicillin and ciprofloxacin (50% and 33.3%, respectively), but 100% sensitivity to aminoglycosides, linezolid, teicoplanin, and quinupristin/dalfopristin. MR-CoNS, though less frequently isolated (N=1 early, N=3 late), displayed uniform susceptibility to tetracyclines, aminoglycosides, rifampicin, linezolid, vancomycin, and teicoplanin.

High susceptibility to linezolid, teicoplanin, quinupristin/dalfopristin, and rifampicin was seen in all Gram-positive isolates in early and late VAP. These are, therefore, effective therapeutic choices in cases of VAP. Vancomycin susceptibility was also ubiquitous in* S. aureus* and MR-CONS, albeit it dropped to 66.6% in late-onset *Enterococcus* infections. The antimicrobial susceptibility profile of Gram-negative isolates (Table 3).

**Table 3:** Susceptibility pattern of Gram-negative organism among early v/s late VAP.

Organisms	*Acinetobacter baumannii* (N=40)		*Klebsiella pneumoniae *(N=29)		*Pseudomonas aeruginosa* (N=15)		*Enterobacter* spp (N=0)		*Citrobacter freundii* (N=2)		
Antibiotics	Early VAP	Late VAP	Early VAP	Late VAP	Early VAP	Late VAP	Early VAP	Late VAP	Early VAP	Late VAP	
Polymyxin B	40 (100%)	39 (100%)	29 (100%)	20 (100%)	15 (100%)	19 (100%)	-	4 (100%)	2 (100%)	4 (100%)	
Colistin	40 (100%)	39 (100%)	29 (100%)	20 (100%)	15 (100%)	19 (100%)	-	4 (100%)	2 (100%)	4(100%)	
Piperacillin	5 (12.5%)	3 (7.6%)	3 (10.3%)	5 (25%)	2 (13.3%)	2 (10.5%)	-	1 (25%)	0	0	
Piperacillin- tazobactam	9 (22.5%)	6 (15.3%)	5 (17.2%)	7 (35%)	8 (53.3%)	4 (21%)	-	2 (50%)	0	3 (75%)	
Amoxiclav	8 (20%)	4 (10.2%)	2 (6.8%)	0	-	2 (10.5%)	-	2(50%)	0	1 (25%)	
Tetracycline	6 (15%)	7 (17.9%)	6 (20.6%)	-	-	4 (21%)	-	2 (50%)	0	1 (25%)	
Ciprofloxacin	5 (12.5%)	5 (12.8%)	2 (6.8%)	4 (20%)	4 (26.6%)	2 (10.5%)	-	1 (25%)	0	0	
Ofloxacin	4 (10%)	5 (12.8%)	2 (6.8%)	4 (20%)	4 (26.6%)	2 (10.5%)	-	1 (25%)	0	0	
Cefepime	7 (17.5%)	6 (15.3%)	5 (17.2%)	5 (25%)	7 (46.6%)	3 (15.7%)	-	2 (50%)	0	1 (25%)	
Amikacin	12 (30%)	22 (56.4%)	18 (62%)	13 (65%)	10 (66.6%)	12 (60.31%)	-	2 (50%)	0	2 (50%)	
Meropenem	22 (55%)	28 (71.7%)	5 (17.2%)	12 (60%)	10 (66.6%)	6 (31.5%)	-	3 (75%)	2 (100%)	2 (50%)	
Aztreonam	8 (20%)	6 (15.3%)	3 (10.3%)	5 (25%)	8 (53.3%)	4 (21%)	-	2 (50%)	0	1 (25%)	

*Acinetobacter baumannii* demonstrated low susceptibility to most agents, although higher sensitivity was noted in late-onset isolates for Amikacin (56.4%) and Meropenem (71.7%). *Klebsiella pneumoniae* showed moderate susceptibility to Amikacin (62-65%) and a marked increase in Meropenem sensitivity in late-onset VAP (60%). Pseudomonas aeruginosa exhibited variable susceptibility, highest for Amikacin (66.6%) and Meropenem (66.6%) in early VAP, with reduced carbapenem sensitivity in late VAP. *Citrobacter freundii*, though infrequent, remained uniformly susceptible to polymyxins and showed moderate sensitivity to carbapenems and β-lactam/β-lactamase inhibitors.

**Figure 1:**
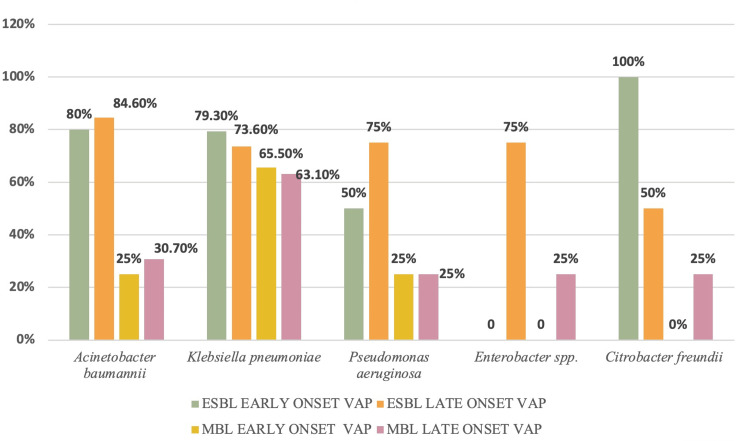
ESBL/MBL profiling of pathogens in early/late VAP.

The prevalence of β-lactamase-producing Gram-negative bacilli (GNB) among early- and late-onset ventilator-associated pneumonia (VAP) isolates is presented in Figure 1.

These findings underscore a high burden of β-lactamase production among GNB in both early and late VAP, with *Klebsiella pneumoniae* showing the greatest propensity for MBL production, while ESBLs were widespread across *Acinetobacter baumannii* and *Klebsiella pneumoniae.*

**Figure 2:**
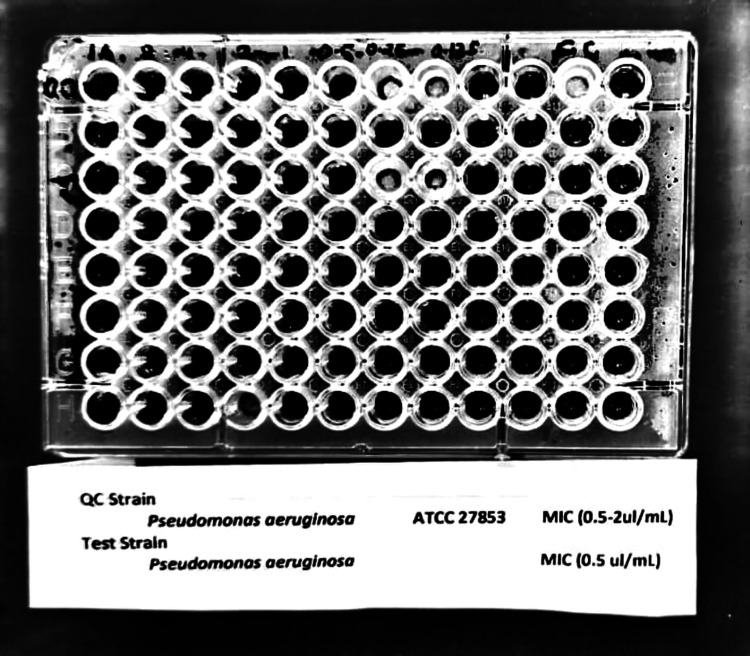
Colistin susceptibility testing by broth microdilution for Pseudomonas aeruginosa

The antimicrobial susceptibility profile of Gram-negative isolates (Table 3) in early and late-onset VAP revealed extensive multidrug resistance, with polymyxin B and colistin showing 100% susceptibility across all species. The quality control strain, *Pseudomonas aeruginosa* (ATCC 27853), showed an MIC within the acceptable range (0.5-2 µg/mL). The test isolate demonstrated an MIC of 0.5 µg/mL, indicating susceptibility (Figure 2).

## Discussion

Our research highlights that, particularly within resource-limited healthcare settings where antibiotic resistance is highly prevalent, VAP remains a major contributor to patient morbidity and mortality. A VAP incidence density of 33.6 per 1,000 ventilator days in our study is consistent with earlier Indian reports (28-35 per 1,000 ventilator-days in tertiary ICUs) but significantly higher (several-fold) than the typical rates (5-10 per 1,000 ventilator-days) observed in Western ICUs, where adherence to VAP prevention bundles is generally better [11,12]. Interestingly, the study found no significant correlation between VAP onset and factors like patient age, gender, or primary diagnosis [13,14,15].

In the microbiological analysis, Gram-negative organisms predominated. The most frequently isolated pathogens were Acinetobacter baumannii, Klebsiella pneumoniae, and Pseudomonas aeruginosa, a distribution consistent with findings from other Indian ICU-based studies [16]. *A. baumannii *was the single most common causative agent, accounting for over 40% of VAP cases, which contrasts with European and North American studies that frequently cite *P. aeruginosa* and MRSA as the main pathogens. This level of resistance is consistent with contemporary Indian ICU studies reporting carbapenem resistance rates in* A. baumanii* ranging from 40-75% [17]. Global ICU surveillance data similarly demonstrate elevated carbapenem resistance in VAP isolates [3].

Profound challenges are revealed in the antibiotic susceptibility pattern. All Gram-negative isolates demonstrated susceptibility to polymyxin B and colistin, antibiotics often considered last-resort measures. *A. baumannii* and *K. pneumoniae,* which are among the key pathogens, show significant resistance to other antibiotics, including carbapenems, aminoglycosides (like amikacin), beta-lactams, and fluoroquinolones [18-21]. Recent Indian and regional surveillance studies report similar carbapenem resistance in *A. baumannii* in ICU settings [18]. Furthermore, the study confirmed that early-onset VAP cases were more likely to involve multidrug-resistant organisms, a finding potentially linked to rapid colonization after broad-spectrum antibiotic use [15,22]. However, the data also offer a slight hope: newer beta-lactam and beta-lactamase inhibitor combinations, such as ceftazidime-avibactam and cefiderocol, show promise against carbapenem-resistant strains [22].

Limitations

This study has several limitations that should be acknowledged. The efficiency of the study could have been improved with a larger sample size. Additionally, as patient follow-up after treatment was not included, the impact of the Antimicrobial Stewardship Program (AMSP) on clinical outcomes could not be assessed.

## Conclusions

In conclusion, the high incidence of VAP and the widespread prevalence of multidrug-resistant Gram-negative organisms highlight the critical and urgent need for comprehensive antimicrobial stewardship, enhanced resistance surveillance, and the integration of VAP prevention bundles (like ventilator care bundles) to minimize exposure and transmission. Tailoring therapy to pathogen-specific susceptibilities not only improves clinical outcomes but also helps curb the emergence of further resistance. Early de-escalation of therapy based on culture results remains paramount.

## References

[1] Kalil AC, Metersky ML, Klompas M (2016). Management of adults with hospital-acquired and ventilator-associated pneumonia: 2016 clinical practice guidelines by the infectious diseases society of America and the American Thoracic Society. Clin Infect Dis.

[2] Torres A, Niederman MS, Chastre J (2017). International ERS/ESICM/ESCMID/ALAT guidelines for the management of hospital-acquired pneumonia and ventilator-associated pneumonia: Guidelines for the management of hospital-acquired pneumonia (HAP)/ventilator-associated pneumonia (VAP) of the European Respiratory Society (ERS), European Society of Intensive Care Medicine (ESICM), European Society of Clinical Microbiology and Infectious Diseases (ESCMID) and Asociación Latinoamericana del Tórax (ALAT). Eur Respir J.

[3] Li W, Cai J, Ding L, Chen Y, Wang X, Xu H (2024). Incidence and risk factors of ventilator-associated pneumonia in the intensive care unit: a systematic review and meta-analysis. J Thorac Dis.

[4] Goel V, Gupta S, Bisht D, Sharma R (2019). Bundle of care approach to reduce ventilator-associated pneumonia in the intensive care unit in a tertiary care teaching hospital in North India. Lung India.

[5] Chawla R (2008). Epidemiology, etiology, and diagnosis of hospital-acquired pneumonia and ventilator-associated pneumonia in Asian countries. Am J Infect Control.

[6] Lim WS (2022). Pneumonia-overview. Encyclopedia of respiratory medicine.

[7] Pilmis B, Zahar JR (2018). Ventilator-associated pneumonia related to ESBL-producing gram negative bacilli. Ann Transl Med.

[8] Alnimr A (2023). Antimicrobial resistance in ventilator-associated pneumonia: predictive microbiology and evidence-based therapy. Infect Dis Ther.

[9] AbdelHalim MM, El Sherbini SA, Ahmed ES, Gharib HA, Elgendy MO, Ibrahim AR, Abdel Aziz HS (2024). Management of ventilator-associated pneumonia caused by Pseudomonas and Acinetobacter organisms in a pediatric center: a randomized controlled study. Medicina (Kaunas).

[10] Gunalan A, Sastry AS, Ramanathan V, Sistla S (2023). Early- vs late-onset ventilator-associated pneumonia in critically ill adults: comparison of risk factors, outcome, and microbial profile. Indian J Crit Care Med.

[11] Mehta Y, Jaggi N, Rosenthal VD (2016). Device-associated infection rates in 20 cities of india, data summary for 2004-2013: findings of the international nosocomial infection control consortium. Infect Control Hosp Epidemiol.

[12] Natarajan K, Bahulikar A, Phalgune DS (2025). Ventilator-associated pneumonia: a prospective observational study. J Assoc Physicians India.

[13] Patel U, Agrawal R, Patel R (2025). Pneumonia in ventilated and non-ventilated patients admitted to intensive care unit in a tertiary care hospital. Lung India.

[14] Dudeck MA, Edwards JR, Allen-Bridson K (2015). National Healthcare Safety Network report, data summary for 2013, Device-associated Module. Am J Infect Control.

[15] Mukhopadhyay H, Bairagi A, Mukherjee A, Prasad AK, Roy AD, Nayak A (2025). Multidrug resistant Acinetobacter baumannii: A study on its pathogenesis and therapeutics. Curr Res Microb Sci.

[16] Meyer E, Schwab F, Gastmeier P (2010). Nosocomial methicillin resistant Staphylococcus aureus pneumonia-epidemiology and trends based on data of a network of 586 German ICUs (2005-2009). Eur J Med Res.

[17] El-Sayed Ahmed MA, Zhong LL, Shen C, Yang Y, Doi Y, Tian GB (2020). Colistin and its role in the era of antibiotic resistance: an extended review (2000-2019). Emerg Microbes Infect.

[18] Chandra S, Prithvi PP, Srija K, Jauhari S, Grover A (2020). Antimicrobial resistance: call for rational antibiotics practice in India. J Family Med Prim Care.

[19] Veeraraghavan B, Shankar C, Karunasree S, Kumari S, Ravi R, Ralph R (2017). Carbapenem resistant Klebsiella pneumoniae isolated from bloodstream infection: Indian experience. Pathog Glob Health.

[20] Saha Saha, P., Chakrabarty Chakrabarty, M., Kabir Kabir (2023). Multidrug Resistance Pattern of Pseudomonas aeruginosa Isolated from Patients with Nosocomial Infection. Ban J Mic.

[21] Arayasukawat P, So-Ngern A, Reechaipichitkul W, Chumpangern W, Arunsurat I, Ratanawatkul P, Chuennok W (2021). Microorganisms and clinical outcomes of early- and late-onset ventilator-associated pneumonia at Srinagarind Hospital, a tertiary center in Northeastern Thailand. BMC Pulm Med.

[22] Montero MM, Domene-Ochoa S, Prim N (2025). Addressing carbapenemase-producing extensively drug-resistant Pseudomonas aeruginosa: the potential of cefiderocol and ceftazidime/avibactam plus aztreonam therapy. Eur J Clin Microbiol Infect Dis.

